# Graphene FET Array Biosensor Based on ssDNA Aptamer for Ultrasensitive Hg^2+^ Detection in Environmental Pollutants

**DOI:** 10.3389/fchem.2018.00333

**Published:** 2018-08-14

**Authors:** Jiawei Tu, Ying Gan, Tao Liang, Qiongwen Hu, Qian Wang, Tianling Ren, Qiyong Sun, Hao Wan, Ping Wang

**Affiliations:** ^1^Biosensor National Special Laboratory, Key Laboratory for Biomedical Engineering of Ministry of Education, Department of Biomedical Engineering, Zhejiang University, Hangzhou, China; ^2^Tsinghua National Laboratory for Information Science and Technology, Tsinghua University, Beijing, China; ^3^Key Laboratory of Healthy & Intelligent Kitchen System Integration of Zhejiang Province, Ningbo, China; ^4^Ningbo Fotile Kitchenware CO. LTD., Bodi Centre, Hangzhou, China

**Keywords:** biosensor, graphene FET array, Hg^2+^ detection, ssDNA aptamer, ultrasensitive detection

## Abstract

Invisible mercury ion is an extremely poisonous environmental pollutant, therefore, a fast and highly sensitive detection method is of significant importance. In this study, a liquid-gated graphene field-effect transistor (GFET) array biosensor (6 × 6 GFETs on the chip) was fabricated and applied for Hg^2+^ quantitate detection based on single-stranded DNA (ssDNA) aptamer. The biosensor showed outstanding selectivity to Hg^2+^ in mixed solutions containing various metal ions. Moreover, the sensing capability of the biosensor was demonstrated by real-time responses and showed a fairly low detection limit of 40 pM, a wide detection ranged from 100 pM to 100 nM and rapid response time below one second. These results suggest that the GFET array biosensor based on ssDNA aptamer offers a simple fabrication procedure and quite fast method for mercury ion contaminant detection and are promising for various analytical applications.

## Introduction

Graphene is a two-dimensional and one-atom thick sheet of sp^2^ hybridized carbon with exceptional electrical and physical properties, such as large detection area, ultra-high electron mobility, tunable ambipolar field-effect characteristic, and biocompatibility, compared to ones based on conventional semiconductor materials. In consequence, graphene field-effect transistor (GFET) have recently attracted much interest in sensing field (Dan et al., 2009; He et al., 2010, 2012; Huang et al., 2010, 2011; Myung et al., 2011; Kim et al., 2015; Han et al., 2017; Li et al., 2017; Kotlowski et al., 2018; Mansouri Majd and Salimi, 2018; Xu et al., 2018) for detection of DNA, protein, ions and so on. Since the first demonstrations, much progress has been achieved in the fabrication and performance of GFETs. In the sensing field, DNA aptamers combining with electrolyte-gated GFET sensor are a preferable choice, since they are smaller in size than Debye length (Maehashi et al., 2007). Therefore, the binding event between the aptamers and the targets can occur within the electric double layer in buffer solution, and as a consequence, changes in the charge distribution with proximity to the graphene can easily be detected (Guo et al., 2005; So et al., 2005). Moreover, the density of the immobilized DNA on the graphene can be controlled, and a high density of DNA can easily be prepared.

Mercury ions (Hg^2+^) are extremely toxic environmental pollutants, which would affect the immune and nervous systems, alter genetic expression, and cause serious damage to both mammals' health and the environment even at very low concentrations (Clarkson et al., 2003). Thus, a fast and highly sensitive Hg^2+^ detection method is of great importance in the healthcare and environmental fields. Several methods have been developed for mercury sensing, including photoelectrochemical methods (Ha et al., 2011), colorimetric analysis (Kim et al., 2012), and oligonucleotide-based sensing (Ono and Togashi, 2004). Therein, biosensing offers considerable advantages including fast response, requirement of low cost, simple equipment, and high sensitivity and selectivity. Recently, much attention has been extended to the development of optical and electrochemical techniques based on DNA for the selective Hg^2+^detection. For example, Liu et al. (2009) developed a novel sensor for Hg^2+^ detection based on a conformational switch using ferrocene-tagged poly-thymine oligonucleotide to form thymine–Hg^2+^-thymine (T–Hg^2+^-T). A report by Zhuang et al. (2013) presented an Hg^2+^ electrochemical biosensor, which used DNA hairpins as recognition elements and exhibited a detection limit of 2.5 nM. Electrochemical strategies for mercury detection based on T–Hg^2+^-T coordination chemistry have been reported by Wang et al. (2014).

At present, the studies of GFET were basically based on a single sensor (Ohno et al., 2009; An et al., 2013; Mansouri Majd and Salimi, 2018), but single GFET has a low current that increases the difficulty of circuit design and signal acquisition. Further, due to the variation of graphene and individual FET, consistent results are hard to be obtained with a single sensor. Therefore, in this study, a common-source array design was used to obtain a large current response and reduce the impact of individual differences. To the best of our knowledge, no study based on GFET array was reported for Hg^2+^ detection with the modification of ssDNA.

Herein, a novel biosensor for Hg^2+^ ultrasensitive determination was designed and fabricated based on common-source graphene field-effect transistors array (6 × 6 GFETs) and ssDNA aptamer. The performance of biosensor was explored by analyzing the Raman spectrum, electrical properties, interferences, and so on. Furthermore, the GFET array modified by ssDNA sequences was applied to Hg^2+^ quantitative determination, which provided a promising method for fast and highly sensitive detection of Hg^2+^ in a variety of applications.

## Methods

### System and materials

**Figure 1:**
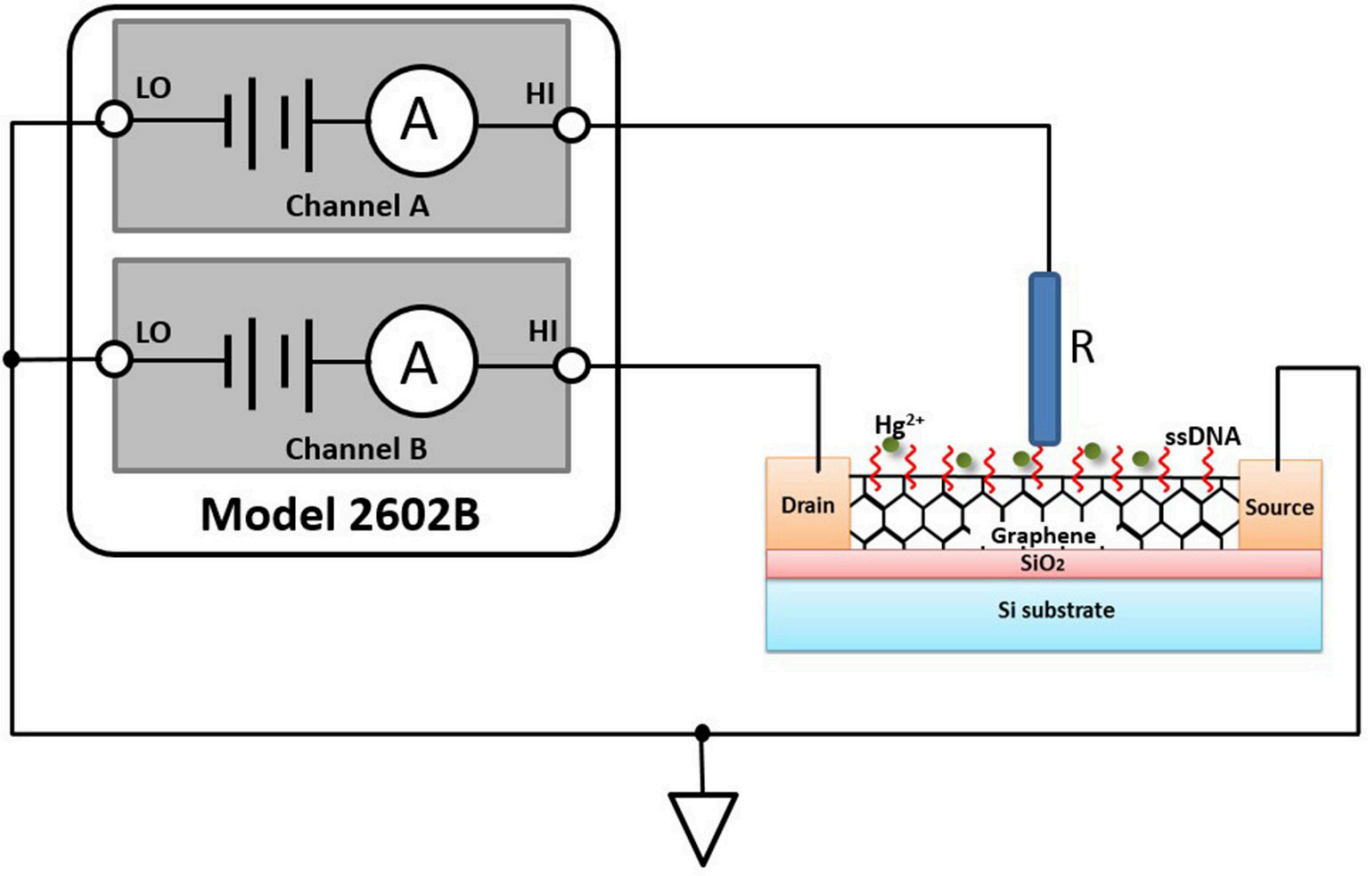
GFET sensor test configuration using multiple SMUs, Model 2602B: HI is short for Force HI terminal, and LO is short for Force LO terminal. R in the figure is an external Ag/AgCl reference electrode with saturated KCl.

Characterizing GFET array's current-voltage (I–V) parameters is extremely crucial to ensuring that it works properly as a sensor and meets specifications. In general, FET-type sensor characteristics testing involves the use of several kinds of instruments, including a sensitive ammeter, multiple voltage sources, and a voltmeter or an alternative approach, a turnkey semiconductor characterization system, which are either tedious or expensive. In our study, Model 2602B (Keithley Instruments Inc., USA), a two-channel source measurement unit (SMU), was utilized to perform parameter testing on the GFET array sensor. The Model 2602B has current resolution to 0.1 fA and can be current limited to prevent damage to the sensor. Figure 1 illustrated an I–V test configuration for an electrolyte-gated GFET sensor using a two-channel Model 2602B, and the GFET sensor had three main terminals: the electrolyte gate, the drain, and the source. A voltage applied to the gate terminal (V_G_) controlled the resulting drain current (I_DS_) that flowed from the drain to the source terminal. In this configuration, the Force HI terminal of Channel A was connected to the gate of the sensor and the Force HI terminal of Channel B was connected to the drain. And the source terminal of the sensor was connected to the Force LO terminals of both SMU channels. A common I–V test performed on the sensor was the drain family of curves (V_G_-I_DS_). With this test, SMU CHB stepped the drain voltage (V_DS_) with a step of 0.1 V from 0 to 3.0 V while SMU CHA swept the gate voltage (V_G_) from −4.0 to 4.0 V and measured the resulting drain current. An external Ag/AgCl reference electrode (saturated KCl) worked as the electrolyte gate of the sensor, which was connected to the Force HI terminal of Channel A.

Reagents used in the experiment were all of analytical grade. Deionized water (18.4 MΩ·cm) used for cleaning and dilution was prepared by an ultra-pure water system Mili-Q (Millipore, USA). The mercury standard solution (100 μg/mL in 3% HNO_3_) was purchased from the National Research Center for Certified Reference Material, China, and de-ionized water was used to dilute the standard solution into different concentrations. Other reagents were purchased from Aladdin (Shanghai, China) and used as received. Analytical grade copper chloride, lead nitrate, chromium chloride, and cadmium chloride were used to prepare stock solutions of 1,000 mg/L of the four metal ions, which were further diluted to the required concentrations before use. The ssDNA was custom-synthesized by GenScript Corp. (USA) and purified by high-performance liquid chromatography. The anti-Hg^2+^ ssDNA sequence used in this study was as follows: 5′-GTTCTTTCGGCTTTGTTC-3′-C7-amino, which could easily form the T-Hg^2+^-T configuration in the present of Hg^2+^ (Lee et al., 2007; Li et al., 2008). All experiments were carried out at room temperature.

### GFET array fabrication

**Figure 2:**
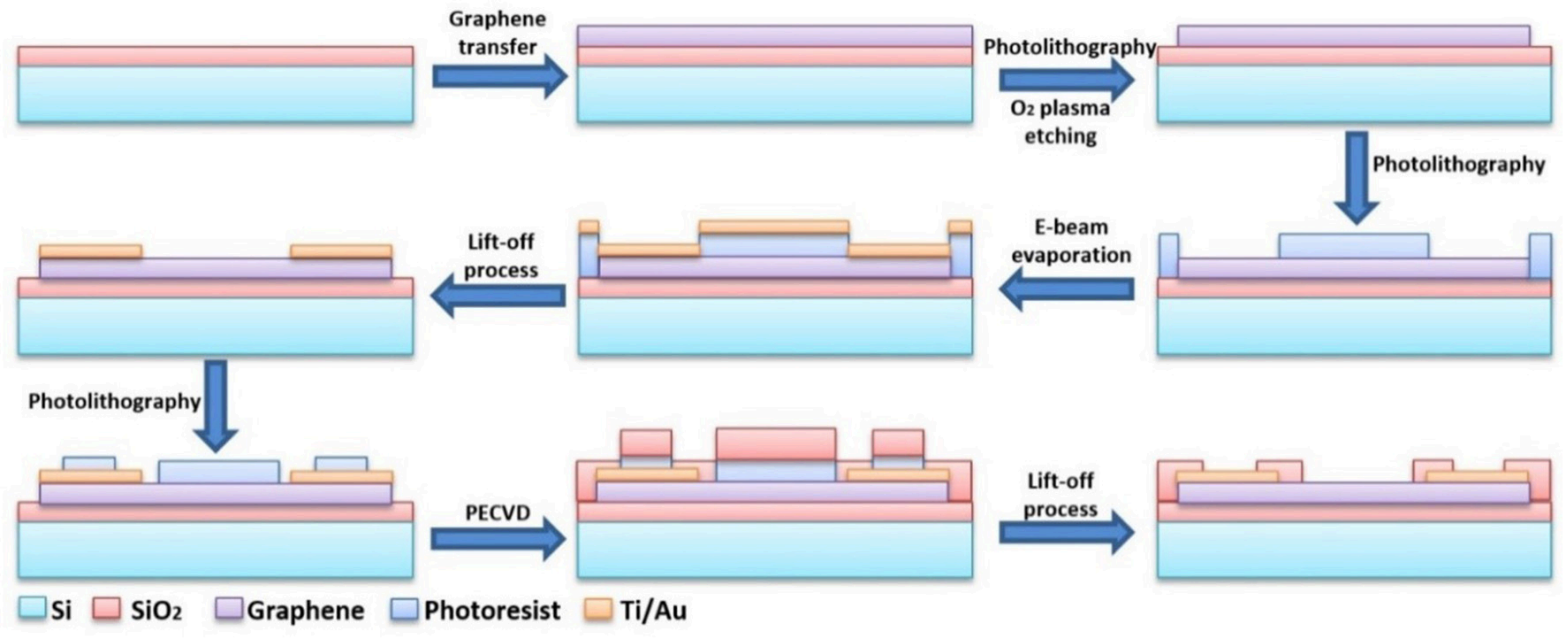
The schematic graphs of the fabrication process flow of the GFET array.

The schematic graphs of the detailed fabrication process flow of the GFET array was shown in Figure 2. The GFET array was fabricated with a series of photolithography methodologies. The graphene film used in the sensor was synthesized by chemical vapor deposition (CVD) on a copper foil and transferred to highly doped silicon substrate with 90 nm thick thermally grown SiO_2_ capping layer. After being spin-coated with polymethylmethacrylate (PMMA), the graphene/Cu film was placed on the surface of the etching solution (1 M FeCl_3_ and 1 M HCl) until the copper foil was completely etched away. And then, the graphene/PMMA film was rinsed by deionized water for several times before transferred onto substrates. PMMA was subsequently removed by acetone. After graphene being transferred, standard photolithography was employed to pattern the channel region of graphene and oxygen plasma etching to remove the unwanted graphene. Next, the metallic layer, which included a titanium layer of 20 nm and a gold layer of 50 nm, was sputtered by e-beam evaporation on the substrate, in which the titanium layer acted as the adhesion layer. And, photolithography was utilized once again to define the shapes of source and drain electrodes followed by lift-off process. Afterwards, a SiO_2_ layer of 100 nm was deposited with plasma enhanced chemical vapor deposition (PECVD) on the metallic layer for insulation. Ultimately, partial insulation areas were etched to expose a designated gold area for spot welding to the external bonding pad and a designed graphene area as the electrolyte gate for the GFET sensor. The final structure of GFET array was seen at the lower right in Figure 2.

**Figure 3:**
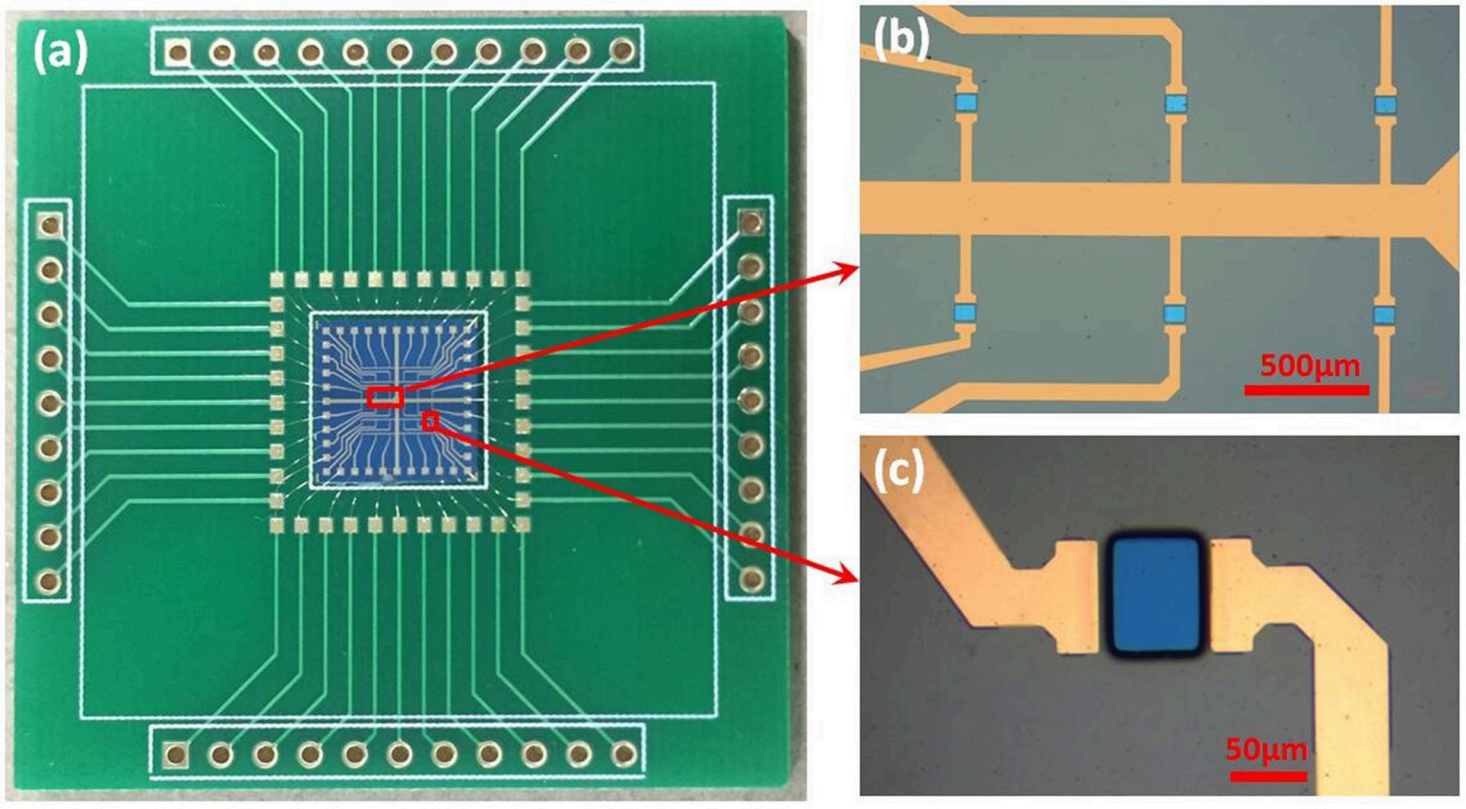
The photograph of the fabricated sensor: **(a)** the packaged sensor with PCB; **(b,c)** the microstructure of GFET array obtained by optical microscopy.

The GFET array was manufactured after all the fabrications mentioned above, the size of which was 9.2 × 9.2 mm. As shown in the Figure 3a, the whole packaged chip of GFET array with printed circuit board (PCB) was 4.2 × 4.2 cm, consisted of 6 × 6 common-source GFETs, with the exposed area of 60 × 80 μm each GFET separated by an 800 μm gap. According to the technical processes above, the graphene was exposed as the electrolyte gate for GFET, just as the blue parts in Figures 3b,c, while the source and drain electrodes (the gold parts in in Figures 3b,c) were covered by 100 nm SiO_2_ as the insulating layer. Figure 3b showed that the 36 GFETs ranked neatly in parallel, which meant all the 36 GFETs shared one source electrode. Finally, the bonding pads of GFET array chip were connected to PCB pads by gold wires and a cell was used in chip encapsulation.

### Fabrication of biosensor

To utilize the GFET array as an ultra-sensitive sensor for Hg^2+^ detection, DNA aptamer with plentiful thymine was functionally attached to the graphene film surface to selectively determine the Hg^2+^. According to the literatures (Chen et al., 2001; Kwon et al., 2012; Park et al., 2012; An et al., 2013), one of the most efficient condensing agents, 1,5-diaminonaphthalene (DAN), was stacked on the side plane of the graphene by π-π interactions between the naphthalene group of DAN and its sp^2^-carbon plane. Hence, the GFET array was treated with 10 μM DAN in methanol (40 μL) for 3 h at room temperature and then flushed with 0.01 M phosphate buffer solution (PBS, pH 7.4). After that, 40 μL 2% (v/v) glutaraldehyde (GA) was conjugated DAN through a Schiff-base reaction in pure environments for another 3 h. The Schiff-base reaction proceeded through chemical attachment between the aldehyde group of GA and the amine group of the DAN connected at the graphene (Lee et al., 2006). Subsequently, after flushing the chip with PBS to remove the superfluous GA, 40 μL ssDNA (20 μM in 0.01 M Tris-HCl buffer solution, pH 7.4) was immobilized to the GA on the graphene surface over 6 h at room temperature, which was also based on the Schiff-base reaction. Finally, the desired ssDNA-based biosensor for Hg^2+^ specific detection was fabricated and rinsed with surplus PBS.

## Results and discussion

### Characterization of common-source liquid-gated GFET array

#### Characterization of graphene on the GFET

**Figure 4:**
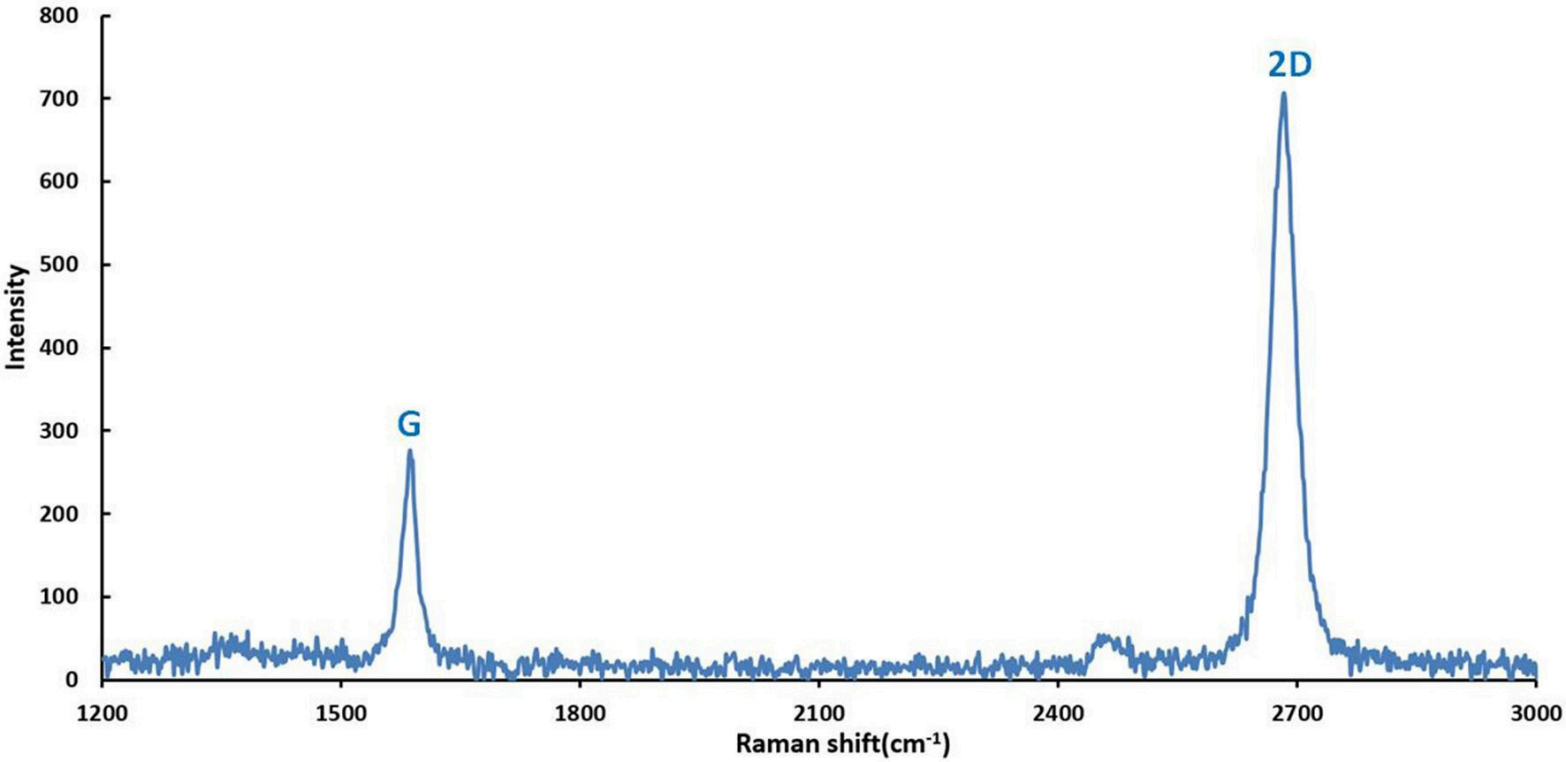
Raman spectrum (514.5 nm laser wavelength) obtained from the graphene film on the sensor showing the G peak and 2D band features characteristic for single-layer graphene (I2D/IG = 2.67).

According to the methods for graphene fabrication described in part 2.2 of this study, graphene film was synthesized by CVD on copper foils and patterned with proper shape after being transferred onto Si/SiO_2_ substrates. Subsequently, the properties of graphene on the surface of GFET array were explored by Raman spectra analysis. The Figure 4 illustrated the Raman spectrum obtained from the graphene film on the GFET array sensor at 514.5 nm laser excitation. The graphene film exhibited typical monolayer graphene features with a sharp G peak and a single 2D peak with a higher intensity. The two prominent peaks appeared at approximate 1,589 and 2,683 cm^−1^, corresponding to G and 2D peaks, respectively. And, the peak intensity ratio of 2.67 between 2D and G confirmed the high percentage of monolayer with fewer multilayer islands in the graphene film on the sensor (Ferrari et al., 2006; Park et al., 2011; Zang et al., 2011; Tian et al., 2012). Meanwhile, the absence of D-band in this spectrum also confirmed the high quality of the as-grown graphene with fewer defects (Graf et al., 2007; Li et al., 2009; Tian et al., 2011).

#### Characterization of common-source GFET array

**Figure 5:**
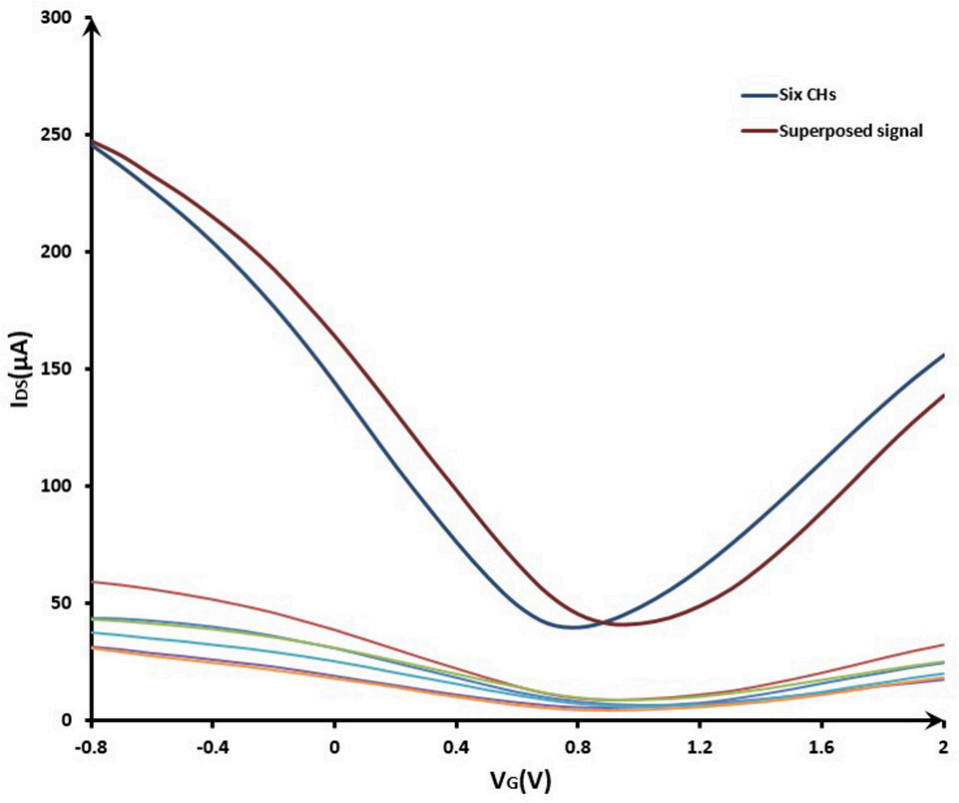
The signal superposition characteristics of the common-source GFET array. VG sweeping from −0.8 to 2.0 V; VDS = 0.5 V.

The signal superposition characteristics of the common-source GFET array was shown in Figure 5. The curves in the lower part of Figure 5 showed the characteristics of six independent GFETs. The red curve in the upper part of Figure 5 was the signal summation of the six independent GFETs. After connecting these six GFETs in parallel, the characteristic curve was shown as the blue curve. It can be seen that the signal intensity of the common-source GFETs had been significantly enhanced compared to a single sensor. The amplitude of superimposed current was the same as the current amplitude in the parallel condition, but the potential shifted because the reference electrode position changed. With the same reason, different single GFET had different distances from the reference electrode, so the characteristic curves of the six independent GFETs were also different. Therefore, in order to increase the response current, the array structure was effective.

#### Characterization of liquid-gated GFET array

**Figure 6:**
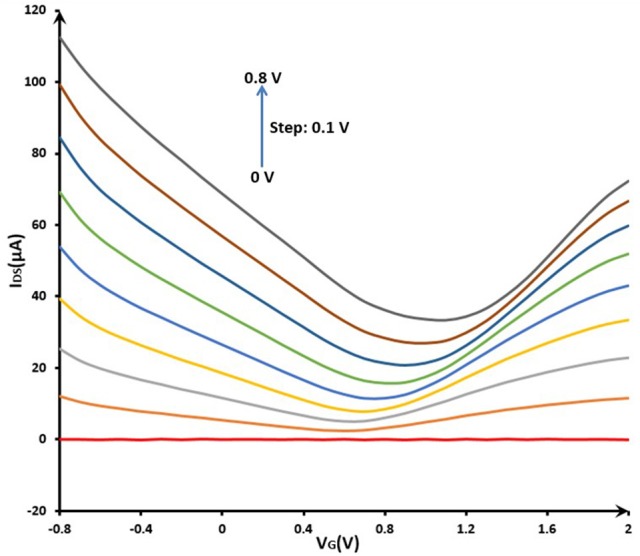
Electrical properties (IDS-VG curve) of GFET array device before modification in phosphate buffer solution (0.01 M PBS, pH 7.4): VG sweeping from −0.8 to 2.0 V; VDS changing from 0 to 0.8 V with a step of 0.1 V.

For the capability test of GFET array, the electrical properties of the liquid-gated GFET array were investigated in the 0.01 M PBS (pH 7.4) at room temperature. The electrical measurements were conducted by the multiple SMUs system described above. The Figure 6 showed that gate voltage (V_G_) was swept from −0.8 to 2.0 V and the resulting drain current was measured at a gradient changing drain voltage (V_DS_) with s step of 0.1 V from 0 to 0.8 V. With increasing gate bias using the reference electrode, the source-drain current decreased in the beginning (V_G_ < 0.8 V), and then increased, which indicated significant bipolar characteristics in blank PBS. The I_DS_-V_G_ curve of GFET array device showed a remarkable Dirac point, which illustrated the structural integrity of common-source graphene FET array sensor. Meanwhile, a suitable quiescent bias point should be considered very cautiously in order to obtain a high dynamic range and steady response for the sensor application. In this way, according to the I_DS_-V_G_ curves, this point was expected to be located around V_DS_ = 0.4~0.8 V and V_G_ = −0.8~−0.4 V for the present device.

### Analytical figures of merit of the GFET biosensor array

**Figure 7:**
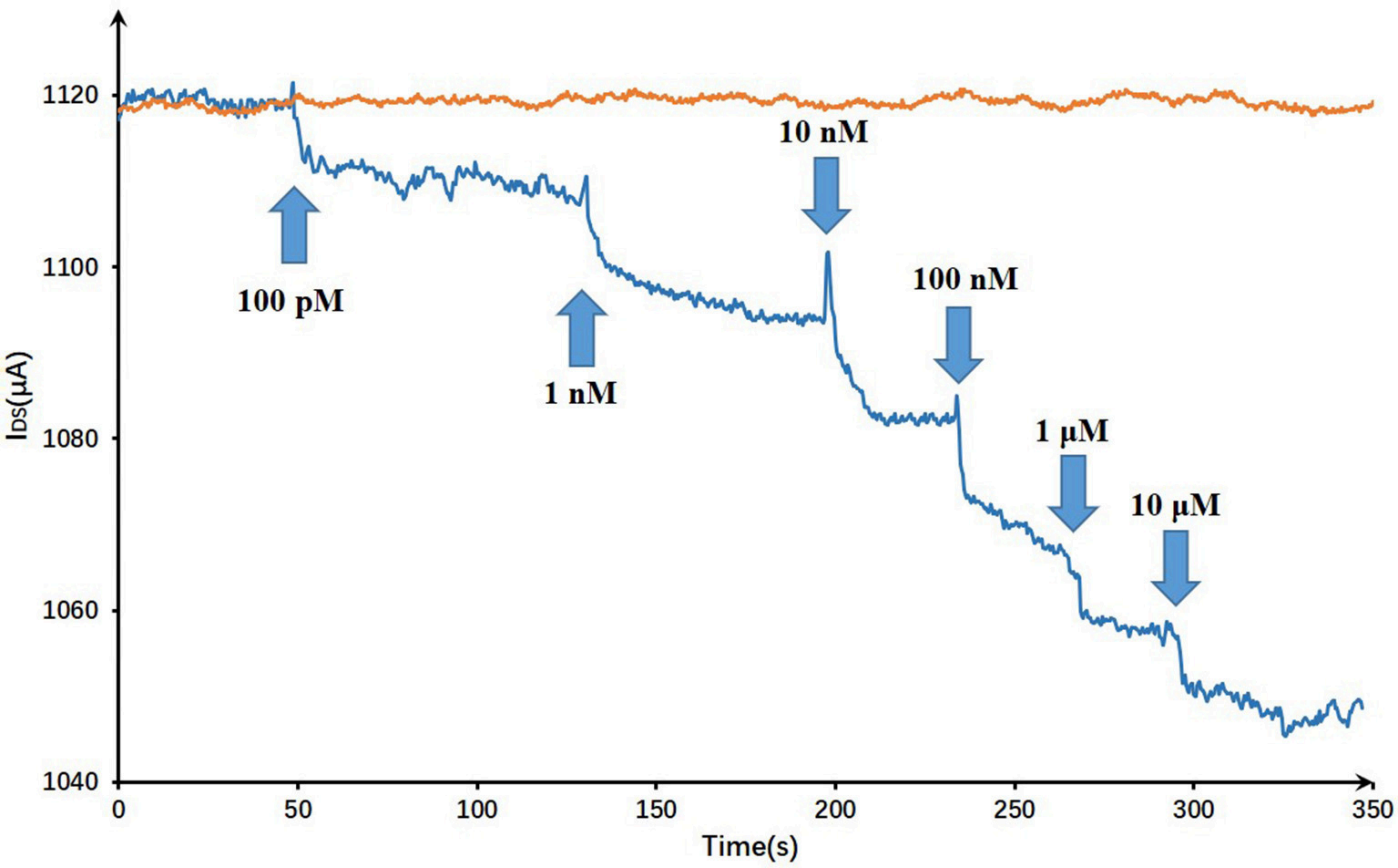
Dynamic responses of the DNA-based biosensor to Hg^2+^ and a blank control: the arrows pointed the adding time and corresponding concentrations.

**Figure 8:**
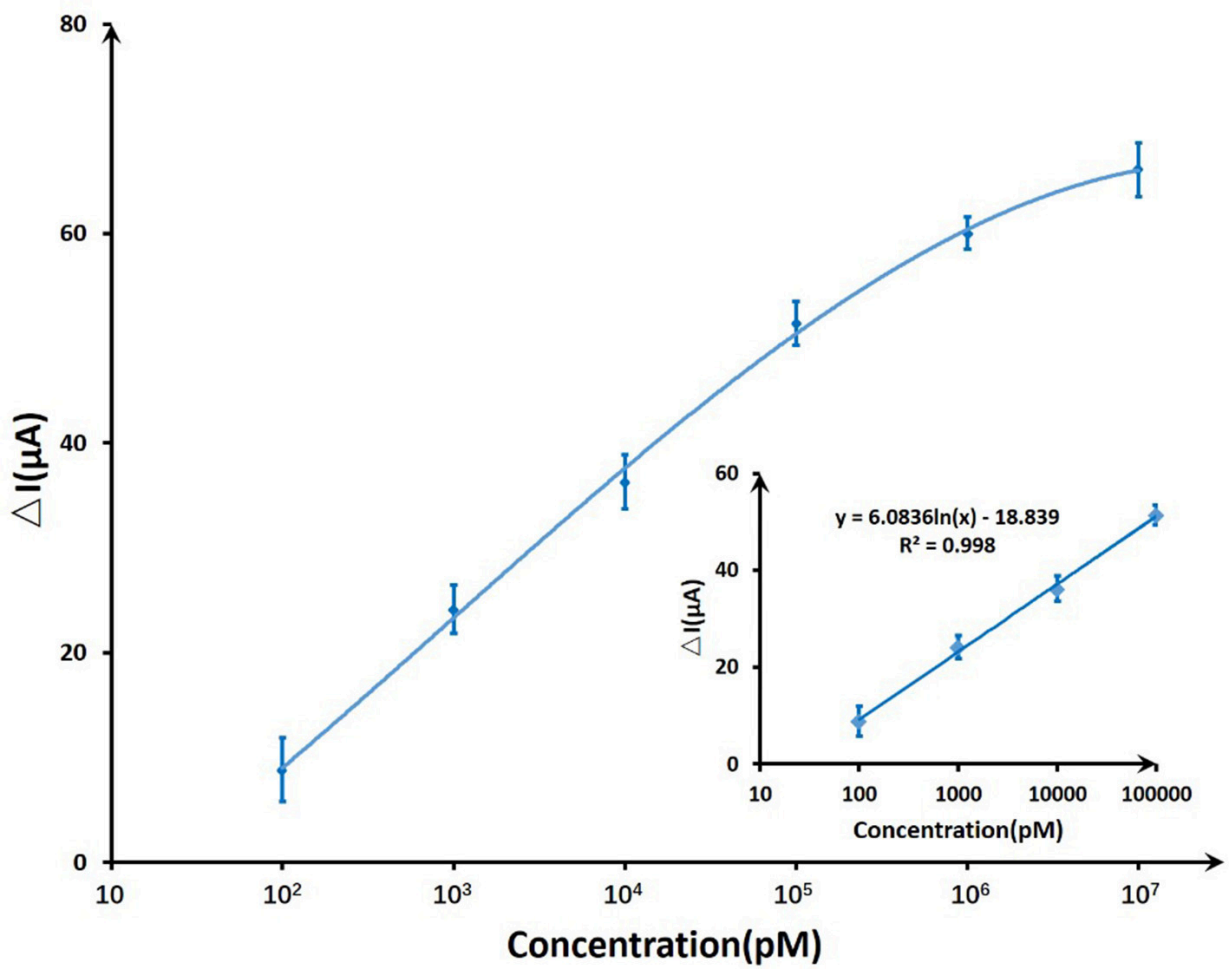
The sensitivity trendline of DNA-based GFET array biosensor to mercury ions ranging from 100 pM to 10 μM. A linear fit was given for the first four concentrations.

On account of T-Hg^2+^-T reaction, Hg^2+^ ions binding to the ssDNA aptamer results electrostatic change at the interface, which will induce a change in the electrical signals. The biosensor performance was demonstrated by measuring I_DS_ upon the addition of various mercury concentrations at a constant V_G_ (V_G_ = −0.8 V) and V_DS_ (V_DS_ = 0.5 V). Figure 7 displayed the real-time responses of GFET biosensor with ssDNA aptamer to different Hg^2+^ concentrations, and I_DS_ gradually decreased when exposed to higher concentrations of Hg^2+^ within concentrations ranging from 100 pM to 10 μM. Moreover, the response time of the GFET array biosensor in the Hg^2+^ determination was pretty fast (on a time scale of <1 s). The biosensor showed a concentration-dependent decrease in I_DS_ when it was exposed to the target Hg^2+^, and in order to further characterize the sensitivity of the ssDNA-based biosensor, the correlation between drain-source current and the logarithm of Hg^2+^ concentration [ln(con)] was investigated as shown in Figure 8. The standard deviations (SDs) in all concentrations were calculated ranging from ±1.54 to ±3.02 μA, and the largest relative standard deviation (RSD) in all concentrations is 9.1%. This low RSD indicates very good repeatability of this biosensor for mercury detection. The current variation increased from 100 pM to 10 μM of the Hg^2+^ concentration. The saturation of the sensor was observed over 1 μM, so the biosensor was calibrated based the current variation of the first four samples. According to the fitting results, the correlation index (*R*^2^) between I_DS_ and ln(con) was 0.998, which showed a quite high correlation between them. Furthermore, the GFET biosensor array exhibited a detection limit of about 40 pM (RSD = 48.64%), which could meet the requirements for Hg^2+^ applications.

**Figure 9:**
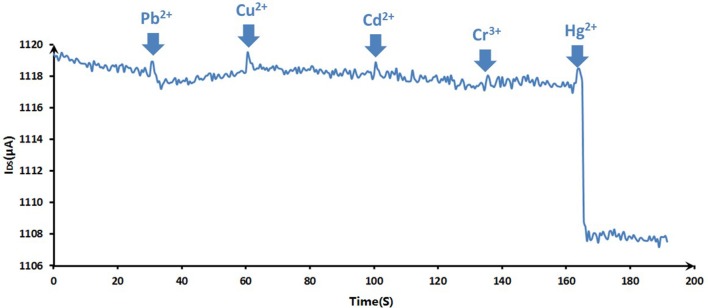
The selective responses of the DNA-based biosensor to various metal ions: Cu^2+^, Pb^2+^, Cr^3+^, Cd^2+^ (10 nM for previous ions) and Hg^2+^ (100 pM). The arrows pointed the adding time for corresponding ions.

The purpose of the experiments wanted to investigate the interference factors for the Hg^2+^ measurements. To evaluate the selectivity of the sensing system for Hg^2+^ ion, the responses of the ssDNA-based biosensor to various suspicious interfering metal ions, which were added in blank 0.01 M PBS (pH 7.4) as analytical samples, were recorded. As Figure 9 showed, Cu^2+^, Pb^2+^, Cr^3+^, Cd^2+^ of 10 nM and Hg^2+^ of 100 pM were added successively in 0.01 M PBS, and the selective responses of GFET array biosensor were recorded for 200 s. From 0 to 160 s, the responses had no obvious change until the addition of Hg^2+^ even in the case that the concentrations of competing ions were a hundred times greater than Hg ion. 100 pM Hg^2+^ could induce a significant current reduction of the biosensor, indicating that the biosensor exhibits a high selectivity to Hg^2+^ over other competing ones.

### Comparison

Compared to other methods and sensors for Hg^2+^ detection, this GFET biosensor array presents high performance (Table 1). The first two studies are based on FET to detect Hg^2+^, and the third study used electrochemical impedance spectroscopy (EIS) measurement based on the T-Hg^2+^-T principle. The fourth work used electrochemical detection for Hg^2+^ based on Au microelectrode array. Compared to other sensors, this ssDNA aptamer GFET array presents wider detection range from 0.1 to 100 nM and the lowest detection limit of 40 pM.

**Table 1:** Comparison of Hg^2+^ detection methods and sensors.

**Detection method**	**Sensor**	**Detection range**	**Detection limit**	**References**
Gate voltage	Alkanethiol modified GFET	Not mentioned	10 ppm (50 nM)	Zhang et al., 2010
Gate voltage	MoS_2_/DNA-Au NPs FET	0.1–10 nM	0.1 nM	Zhou et al., 2016
EIS	Gold disk electrode	0.1nM−10 μM	0.1 nM	Lin et al., 2011
Electrochemical	Au microelectrode array	10–100 nM	4 nM	Huan et al., 2012
Gate voltage	ssDNA aptamer GFET array	0.1–100 nM	40 pM	This work

Due to the very tight bonding between T-Hg^2+^-T, the current response would keep decreasing when bonding with more Hg^2+^. Thus, the response baseline would keep changing during the stability test, which is hard to be evaluated. The biosensor can be recycled before the saturation of the biosensor. Between the two measurements, the biosensor should be kept in 4°C to maintain the performance of the biosensor. When starting the measurement, the biosensor should be washed using PBS and measured in a blank sample to obtain a stable baseline. Then the biosensor can be used for the following tests.

For detecting Hg^2+^, the GFET array is highly sensitive, and capable for online and continuous monitoring. However, unlike detection in a buffer, water contaminant detection is usually carried out in a natural water system. In natural environment, mercury may exist as complexes which can have an effect on the results. Moreover, the suspended solid and organic and inorganic materials in water may disturb the GFET signal, leading to large uncertainties during the detection and may reduce the sensitivity and lifetime of the sensor. Therefore, to extend the GFET array into practical applications, further work is needed.

## Conclusion

A liquid-gated GFET array with 36 common-source FETs was fabricated and applied for Hg^2+^ detection as a biosensor decorated with ssDNA aptamer. The characterization of GFET array was tested and discussed based on the Raman spectrum and electrical methods on the multiple SMUs system. The array structure with common-source was effective in increasing the response current. The biosensor showed good selectivity to Hg^2+^ in mixed solutions containing various metal ions. Moreover, the sensing capability of the biosensor was demonstrated by real-time responses and showed a fairly low detection limit of about 40 pM and quite rapid response time below 1 s. Compared with other similar detection methods and sensors, the constructed biosensor presents an excellent detection level. Overall, the simple fabrication procedure and the excellent sensing performance of the GFET array structure make it promising for contaminant detection of Hg^2+^ in water environment.

## Author contributions

JT, QS, TR, and PW conceived the idea and designed the experiments. TL, QW, and TR designed and manufactured sensors. JT, YG, QH, and HW performed experiments. JT, YG, TL, QW, QH, and HW contributed to data analysis and interpretation. JT and PW wrote this paper. All authors discussed the results and commented on the manuscript.

### Conflict of interest statement

The authors declare that the research was conducted in the absence of any commercial or financial relationships that could be construed as a potential conflict of interest.

## References

[B1] An J. H. Park S. J. Kwon O. S. Bae J. Jang J. (2013). High-performance flexible graphene aptasensor for mercury detection in mussels. ACS Nano 7, 10563–10571. 10.1021/nn402702w 24279823

[B2] Chen R. J. Zhang Y. Wang D. Dai H. (2001). Noncovalent sidewall functionalization of single-walled carbon nanotubes for protein immobilization. J. Am. Chem. Soc. 123, 3838–3839. 10.1021/ja010172b 11457124

[B3] Clarkson T. W. Magos L. Myers G. J. (2003). The toxicology of mercury—current exposures and clinical manifestations. N. Engl. J. Med. 349, 1731–1737. 10.1056/NEJMra022471 14585942

[B4] Dan Y. Lu Y. Kybert N. J. Luo Z. Johnson A. T. (2009). Intrinsic response of graphene vapor sensors. Nano Lett. 9, 1472–1475. 10.1021/nl8033637 19267449

[B5] Ferrari A. C. Meyer J. Scardaci V. Casiraghi C. Lazzeri M. Mauri F.. (2006). Raman spectrum of graphene and graphene layers. Phys. Rev. Lett. 97:187401. 10.1103/PhysRevLett.97.187401 17155573

[B6] Graf D. Molitor F. Ensslin K. Stampfer C. Jungen A. Hierold C.. (2007). Spatially resolved Raman spectroscopy of single-and few-layer graphene. Nano Lett. 7, 238–242. 10.1021/nl061702a 17297984

[B7] Guo X. Huang L. O'Brien S. Kim P. Nuckolls C. (2005). Directing and sensing changes in molecular conformation on individual carbon nanotube field effect transistors. J. Am. Chem. Soc. 127, 15045–15047. 10.1021/ja054335y 16248641

[B8] Ha D. Yu H. Hu N. Wu C. Zhou J. Kirsanov D. (2011). “Portable e-tongue based on multi-channel LAPS array with PVC membrane for rapid environment detection,” in AIP Conference Proceedings, AIP (New York, NY), 187–188.

[B9] Han D. Chand R. Kim Y. S. (2017). Microscale loop-mediated isothermal amplification of viral DNA with real-time monitoring on solution-gated graphene FET microchip. Biosens. Bioelectron. 93, 220–225. 10.1016/j.bios.2016.08.115 27623280

[B10] He Q. Sudibya H. G. Yin Z. Wu S. Li H. Boey F.. (2010). Centimeter-long and large-scale micropatterns of reduced graphene oxide films: fabrication and sensing applications. ACS Nano 4, 3201–3208. 10.1021/nn100780v 20441213

[B11] He Q. Wu S. Yin Z. Zhang H. (2012). Graphene-based electronic sensors. Chem. Sci. 3, 1764–1772. 10.1039/c2sc20205k

[B12] Huan T. N. Hung L. Q. Ha V. T. Anh N. H. Van Khai T. Chung H. (2012). Spirally oriented Au microelectrode array sensor for detection of Hg (II). Talanta 94, 284–288. 10.1016/j.talanta.2012.03.041 22608449

[B13] Huang Y. Dong X. Liu Y. Li L.-J. Chen P. (2011). Graphene-based biosensors for detection of bacteria and their metabolic activities. J. Mater. Chem. 21, 12358–12362. 10.1039/c1jm11436k

[B14] Huang Y. Dong X. Shi Y. Li C. M. Li L. J. Chen P. (2010). Nanoelectronic biosensors based on CVD grown graphene. Nanoscale 2, 1485–1488. 10.1039/c0nr00142b 20820739

[B15] Kim H. N. Ren W. X. Kim J. S. Yoon J. (2012). Fluorescent and colorimetric sensors for detection of lead, cadmium, and mercury ions. Chem. Soc. Rev. 41, 3210–3244. 10.1039/C1CS15245A 22184584

[B16] Kim J. Lee M. S. Jeon S. Kim M. Kim S. Kim K.. (2015). Highly transparent and stretchable field-effect transistor sensors using graphene–nanowire hybrid nanostructures. Adv. Mater. 27, 3292–3297. 10.1002/adma.201500710 25885929

[B17] Kotlowski C. Larisika M. Guerin P. M. Kleber C. Kröber T. Mastrogiacomo R. (2018). Fine discrimination of volatile compounds by graphene-immobilized odorant-binding proteins. Sens. Actuat. B 256, 564–572. 10.1016/j.snb.2017.10.093

[B18] Kwon O. S. Park S. J. Hong J. Y. Han A. R. Lee J. S. Jang J. (2012). Flexible FET-type VEGF aptasensor based on nitrogen-doped graphene converted from conducting polymer. ACS Nano 6, 1486–1493. 10.1021/nn204395n 22224587

[B19] Lee J. S. Han M. S. Mirkin C. A. (2007). Colorimetric detection of mercuric ion (Hg2+) in aqueous media using DNA-functionalized gold nanoparticles. Angew. Chem. 119, 4171–4174. 10.1002/ange.200700269 17461429

[B20] Lee L. M. Heimark R. L. Baygents J. C. Zohar Y. (2006). Self-aligned immobilization of proteins utilizing PEG patterns. Nanotechnology 17:S29. 10.1088/0957-4484/17/4/006 21727351

[B21] Li D. Wieckowska A. Willner I. (2008). Optical analysis of Hg2+ ions by oligonucleotide–gold-nanoparticle hybrids and DNA-based machines. Angew. Chem. 120, 3991–3995. 10.1002/ange.200705991 18404745

[B22] Li X. Cai W. An J. Kim S. Nah J. Yang D.. (2009). Large-area synthesis of high-quality and uniform graphene films on copper foils. Science 324, 1312–1314. 10.1126/science.1171245 19423775

[B23] Li Y. Wang C. Zhu Y. Zhou X. Xiang Y. He M.. (2017). Fully integrated graphene electronic biosensor for label-free detection of lead (II) ion based on G-quadruplex structure-switching. Biosens. Bioelectron. 89, 758–763. 10.1016/j.bios.2016.10.061 27816595

[B24] Lin Z. Li X. Kraatz H. B. (2011). Impedimetric immobilized DNA-based sensor for simultaneous detection of Pb2+, Ag+, and Hg2+. Anal. Chem. 83, 6896–6901. 10.1021/ac2014096 21797211

[B25] Liu S. J. Nie H. G. Jiang J. H. Shen G. L. Yu R. Q. (2009). Electrochemical sensor for mercury (II) based on conformational switch mediated by interstrand cooperative coordination. Anal. Chem. 81, 5724–5730. 10.1021/ac900527f 19522530

[B26] Maehashi K. Katsura T. Kerman K. Takamura Y. Matsumoto K. Tamiya E. (2007). Label-free protein biosensor based on aptamer-modified carbon nanotube field-effect transistors. Anal. Chem. 79, 782–787. 10.1021/ac060830g 17222052

[B27] Mansouri Majd S. Salimi A. (2018). Ultrasensitive flexible FET-type aptasensor for CA 125 cancer marker detection based on carboxylated multiwalled carbon nanotubes immobilized onto reduced graphene oxide film. Anal. Chim. Acta 1000, 273–282. 10.1016/j.aca.2017.11.008 29289320

[B28] Myung S. Solanki A. Kim C. Park J. Kim K. S. Lee K. B. (2011). Graphene-encapsulated nanoparticle-based biosensor for the selective detection of cancer biomarkers. Adv. Mater. 23, 2221–2225. 10.1002/adma.201100014 21469221 PMC3181002

[B29] Ohno Y. Maehashi K. Yamashiro Y. Matsumoto K. (2009). Electrolyte-gated graphene field-effect transistors for detecting pH and protein adsorption. Nano Lett. 9, 3318–3322. 10.1021/nl901596m 19637913

[B30] Ono A. Togashi H. (2004). Highly selective oligonucleotide-based sensor for mercury (II) in aqueous solutions. Angew. Chem. Int. Edn. 43 4300–4302. 10.1002/anie.200454172 15368377

[B31] Park J. Xiong W. Gao Y. Qian M. Xie Z. Mitchell M. (2011). Fast growth of graphene patterns by laser direct writing. Appl. Phys. Lett. 98:123109 10.1063/1.3569720

[B32] Park S. J. Kwon O. S. Lee S. H. Song H. S. Park T. H. Jang J. (2012). Ultrasensitive flexible graphene based field-effect transistor (FET)-type bioelectronic nose. Nano Lett. 12, 5082–5090. 10.1021/nl301714x 22962838

[B33] So H. M. Won K. Kim Y. H. Kim B. K. Ryu B. H. Na P. S.. (2005). Single-walled carbon nanotube biosensors using aptamers as molecular recognition elements. J. Am. Chem. Soc. 127, 11906–11907. 10.1021/ja053094r 16117506

[B34] Tian H. Ren T. L. Xie D. Wang Y. F. Zhou C. J. Feng T. T.. (2011). Graphene-on-paper sound source devices. ACS Nano 5, 4878–4885. 10.1021/nn2009535 21591811

[B35] Tian H. Xie D. Yang Y. Ren T. L. Wang Y. F. Zhou C. J.. (2012). Single-layer graphene sound-emitting devices: experiments and modeling. Nanoscale 4, 2272–2277. 10.1039/c2nr11572g 22214995

[B36] Wang W. Wang Y. Tu L. Klein T. Feng Y. Li Q.. (2014). Magnetic detection of mercuric ion using giant magnetoresistance-based biosensing system. Anal. Chem. 86, 3712–3716. 10.1021/ac404015j 24654958

[B37] Xu S. Jiang S. Zhang C. Yue W. Zou Y. Wang G. (2018). Ultrasensitive label-free detection of DNA hybridization by sapphire-based graphene field-effect transistor biosensor. Appl. Surf. Sci. 427, 1114–1119. 10.1016/j.apsusc.2017.09.113

[B38] Zang Y. Xie D. Wu X. Chen Y. Lin Y. Li M. (2011). Enhanced photovoltaic properties in graphene/polycrystalline BiFeO3/Pt heterojunction structure. Appl. Phys. Lett. 99:132904 10.1063/1.3644134

[B39] Zhang T. Cheng Z. Wang Y. Li Z. Wang C. Li Y.. (2010). Self-assembled 1-octadecanethiol monolayers on graphene for mercury detection. Nano Lett. 10, 4738–4741. 10.1021/nl1032556 20931998

[B40] Zhou G. Chang J. Pu H. Shi K. Mao S. Sui X. (2016). Ultrasensitive mercury ion detection using DNA-functionalized molybdenum disulfide nanosheet/gold nanoparticle hybrid field-effect transistor device. ACS Sensors 1, 295–302. 10.1021/acssensors.5b00241

[B41] Zhuang J. Fu L. Tang D. Xu M. Chen G. Yang H. (2013). Target-induced structure-switching DNA hairpins for sensitive electrochemical monitoring of mercury (II). Biosens. Bioelectron. 39, 315–319. 10.1016/j.bios.2012.07.015 22863115

